# COVID-19 infection, vaccine status, and avoidance behaviors in adults with attention deficit and hyperactivity disorder: A cross-sectional study

**DOI:** 10.3389/fpsyt.2022.938111

**Published:** 2022-08-24

**Authors:** Ozge Kilic, Muhammed Emin Boylu, Sila Karakaya-Erdur, Merve Suma-Berberoglu, Gisli Gudjonsson, Susan Young, Erdem Deveci, Ismet Kirpinar

**Affiliations:** ^1^Department of Psychiatry, Faculty of Medicine, Bezmialem Vakif University, Istanbul, Turkey; ^2^Department of Psychiatry, Liv Hospital, Istanbul, Turkey; ^3^Department of Psychology, King's College London, Institute of Psychiatry, Psychology & Neuroscience, London, United Kingdom; ^4^Department of Psychology, Reykjavík University, Reykjavik, Iceland; ^5^Department of Psychiatry, Faculty of Medicine, Istanbul Medipol University, Istanbul, Turkey

**Keywords:** attention deficit disorder, attention deficit hyperactivity disorder (ADHD), COVID-19 vaccine, SARS-CoV-2, behavioral avoidance, risk-mitigation, coronavirus, COVID-19 infection

## Abstract

**Objective:**

We aim to examine infection risk and vaccine status of COVID-19 in attention deficit and hyperactivity disorder and evaluate the impact of demographic, clinical, and COVID-19-related factors on the infection status and behavioral avoidance of COVID-19.

**Methods:**

This cross-sectional study assessed adults with attention deficit and hyperactivity disorder recruited from an outpatient psychiatry clinic. Patients and healthy controls completed a survey on sociodemographic data, COVID-19 infection status, and vaccine status. COVID-19 Disease Perception Scale, COVID-19 Avoidance Attitudes Scale, Attitudes toward COVID-19 Vaccine Scale, Adult Attention Deficit and Hyperactivity Disorder Self-report Screening Scale for DSM-5, Adult Attention Deficit and Hyperactivity Disorder Self-Report Scale Symptoms Checklist, Patient Health Questionnaire-9, and State-Trait Anxiety Inventory were applied.

**Results:**

Ninety patients and 40 healthy controls participated. Patients did not differ from controls in COVID-19 infection and vaccine status, and behavioral avoidance of COVID-19. No demographic and clinical factor significantly affected the COVID-19 infection status. Patients scored higher than controls in the perception of COVID-19 as contagious (*p* = 0.038), cognitive avoidance of COVID-19 (*p* = 0.008), and positive attitudes toward the COVID-19 vaccine (*p* = 0.024). After adjustment of possible factors, a positive perception of the COVID-19 vaccine and a perception of COVID-19 as dangerous were the two factors significantly affecting behavioral avoidance of COVID-19 [*R*^2^ = 0. 17, *F*(2) = 13.189, *p* < 0.0001].

**Conclusion:**

Infection and vaccine status of COVID-19 in patients did not significantly differ from controls. No demographic and clinical factor significantly affected the COVID-19 infection status. Approximately four-fifths of the patients were fully vaccinated as recommended by national and global health organizations. This has increased the knowledge base showing that the COVID-19 vaccine is acceptable and receiving the vaccine is endorsed by ADHD patients. Attention deficit and hyperactivity disorder itself may provoke no kind of mental disturbance in sense of perception of the danger of this disease. Our findings have increased the knowledge base showing that the COVID-19 vaccine is acceptable and the actual practice of receiving the vaccine is endorsed in this population. Our message for practice would be to take into account not only the core symptoms and the comorbidities of the disorder but also the perception of the disease while exploring its link with COVID-19.

## Introduction

Having entered its third year, the COVID-19 pandemic has resulted in 500 million people being infected and more than 6 million deaths by April 2022 according to World Health Organization. Several risk-mitigation behaviors were recommended to reduce the risk of transmission and infection. These included being vaccinated as officially recommended, wearing masks in public places, and avoiding close physical contact with people outside one's household (i.e., social distancing).

Vulnerable people have been identified as those having certain pre-existing medical and mental disorders. Attention-deficit hyperactivity disorder (ADHD) is one of the neurodevelopmental disorders that are on the updated list of the Centers for Disease Control and Prevention (CDC) as high-risk medical conditions for COVID-19 (1).

One of the most critical public health measures in response to COVID-19's prolonged nature has been the significance of risk-mitigation measures in limiting exposure and severe illness. If specific subgroups of the population had a higher risk of COVID-19 infection, considering the highly contagious nature of the coronavirus, targeted and tailored risk-mitigation strategies for these groups may aid in controlling the COVID-19 or other contagious diseases. For now, the available literature has been conflicting and does not consistently show that ADHD patients represent such a specific subgroup of the population. One of the studies that support ADHD is associated with COVID-19 was carried out in Israel from electronic health records of patients aged from 2 months to 103 years. The increased COVID-19 risk was suggested to be higher in untreated ADHD compared to treated ADHD patients (2). Patients with a recent ADHD diagnosis have been reported to have a significantly higher risk of COVID-19 in another study conducted up to July 2020 in the United States of America (3). ADHD was associated with a significantly higher rate of hospitalization and being symptomatic (4). A systematic review and a meta-analysis demonstrated that ADHD patients have increased susceptibility and severity compared to controls (5). However, a recent longitudinal study that investigated pre-pandemic and pandemic data on neurodevelopmental conditions did not show strong evidence of differences in the distribution of infections in those with ADHD compared to those without (6). Other studies failed to show that youth with ADHD were more likely to experience COVID-19 infection compared with non-ADHD peers (7). On the contrary, Rajkumar et al. (8) demonstrated that ADHD prevalence was statistically inversely linked with COVID-19 prevalence after controlling for medical conditions, demographic, climate-related, and economic variables. Other researchers demonstrated that rates of recovery increased with the prevalence of ADHD and proposed ADHD may have evolutionary benefits for managing coronavirus, as opposed to being a risk factor (9).

The fight against the pandemic is highly dependent on individual compliance (10). If a specific group of people has an increased risk of COVID-19, this may relate to an increased risk of infecting other people. For example, youth with the combined presentation of ADHD were shown to fail to comply with hygiene behaviors. Other than hygiene behaviors another risk mitigation factors are avoidance behaviors. We identified only one study that explored avoidance behaviors among the youth of 5–21 ages that has shown no association (11). The most promising method of containing the COVID-19 pandemic is the use of vaccines to prevent SARS-CoV-2 infection. Adolescents with ADHD were reported to have greater hesitancy and less confidence in COVID-19 vaccine safety compared to adolescents without ADHD (12). We could not identify a study that explored the vaccine attitude among adults with ADHD.

Therefore, with this study, we aim to examine: (1) COVID-19 infection and vaccine status in adults with ADHD; (2) factors that affected infection risk; and (3) factors that impact avoidance of COVID-19.

We hypothesized that ADHD patients would exhibit a higher rate of COVID-19 infection and fewer avoidance behaviors compared with controls. They would be equally willing to accept the COVID-19 vaccine. The perception and avoidance of COVID-19, attitudes toward the COVID-19 vaccine, inattention, hyperactivity-impulsivity (HI) anxiety, and depression would impact the behavioral avoidance of COVID-19.

## Methods

### Participants and procedures

This study adopted a cross-sectional design. It was conducted under the Helsinki Declaration of 1975. Ethics committee approval was obtained from the university's non-interventional research ethics committee. Participants' informed consent was obtained prior to the collection of data. To adhere to the epidemic prevention policy, questionnaires were distributed online. No incentive was given to participate in the study. All data collection from patients and controls took place between January 2021 and March 2022.

The study population was derived from the database of previous adult patients who admitted to the psychiatry outpatient clinic of a tertiary university hospital between January 2019 and December 2020. From all patients that were screened, those whose diagnostic codes were recorded in the principal diagnosis field as ADHD were identified. To be included, the patients had to be between 18 and 60 years old, be able to read and write without help, and had been diagnosed with ADHD by a psychiatry specialist with a face-to-face clinical interview according to the Diagnostic and Statistical Manual of Mental Disorders, Fifth Edition (DSM-5) (13). Exclusion criteria for patients were schizophrenia spectrum and other psychotic disorders, dementia, bipolar disorder, and unwillingness to participate. Two hundred sixty-six patients with ADHD that comply with this criteria were identified and contacted by phone. After explaining the purpose of the study, they were invited to complete the online questionnaire which was shared through an online link. The survey was created using Google Forms^®^, a free program. Out of 266 invitations, 60 responses were received with a response rate of 22.6%. There were no significant differences in terms of age, gender, and education between patients that participated and those that did not participate. Additionally, 30 volunteering ADHD patients that have visited and have been diagnosed in the same outpatient clinic completed the online link between January 2021 and March 2022.

For recruitment of the healthy controls, advertisements in the hospital, waiting rooms, and university campus were used. Inclusion criteria for controls were age between 18 and 60 years old, and the ability to read and write without help. Exclusion criteria for controls were being diagnosed with a current psychiatric disorder, being prescribed any psychotropic medication currently, and having an active unstable medical illness. Healthy controls received the same Google form link and completed the survey online.

Patients and controls were matched for sex, education, and income level. The median age of the controls was significantly higher than the patients (**Table 2**). Data from 130 participants (90 patients and 40 healthy controls) were analyzed for the study.

### Outcomes

The main outcomes were behavioral avoidance score and the presence of a history of COVID positive testing. The sources of data for the main outcomes were a subgroup score of Avoidance Attitudes from the COVID-19 scale and the self-report of the patient on COVID testing or not. Primary independent variables of interest were age, the ADHD symptom scores of IN, HI, subscores of COVID-19 Disease Perception, attitude toward COVID-19 vaccine, depression score, and state and trait anxiety scores. Missing data were very few and the mean score of that variable was entered to replace missing data. To prevent the potential source of selection bias, we attempted to recruit all eligible patients diagnosed with ADHD that were in the database, every patient was contacted with three reminders during data collection. The selection of the independent variables was based on the characteristics that could affect COVID-19 infection and behavioral avoidance of it. These were the disorder's clinical presentation, potential comorbidities, and the patients' image of COVID-19 infection and its vaccine. Quantitative variables from three COVID-19 scales and Adult ADHD Self-Report Scale Symptoms Checklist (ASRS v1.1) were handled in two subgroups of quantitative variables because of the different constructs they were representing.

### Data collection tools

The online survey consisted of three parts (i) demographics (sex, age, marital, educational status, vocational, living, and income status), (ii) clinical characteristics (medication, COVID-19 testing, COVID-19 vaccination), and (iii) validated scales. COVID-19 vaccination status was classified as complete, incomplete, or no vaccination concerning the recommendations of the national health authority. Less than two live attenuated vaccines or less than three inactive vaccines were accepted as incomplete vaccination based on the national vaccine administration strategy.

### COVID-19 disease perception scale

Perception of COVID-19 (P-COVID-19) was measured by a valid and reliable scale. The scale consists of seven items and two sub-dimensions: “dangerousness” and “contagiousness” and is in a five-point Likert structure, The expressions are evaluated as “I strongly disagree (1),” “I do not agree (2),” “I am undecided (3),” “I agree (4),” “I strongly agree (5).” Sub-dimension of dangerousness covers perceptions and beliefs about the danger posed by COVID-19. The contagiousness subdimension consists of items related to perceptions of the contagiousness of the disease. Some items in the dangerousness sub-dimension are reversely coded. A value between 1 and 5 is obtained by dividing the total score obtained by summing the item scores in the scale sub-dimension by the number of items in that sub-dimension. High scores in the dangerousness sub-dimension indicate a high perception of the dangerousness of the disease, and high scores in the contagiousness sub-dimension indicate the perception of the contagiousness of the virus. Inverse items 1 → 5; 2 → 4; 3 → 3; 4 → 2; It is encoded as 5 → 1 (10).

### Attitudes toward the COVID-10 vaccine scale

Attitudes toward the COVID-19 Vaccine (ATV-COVID19) scale has 9 items, 4 items for a positive attitude, and 5 items for a negative attitude. The statements in the scale are evaluated as “Strongly disagree (1),” “Disagree (2),” “Undecided (3),” “Agree (4),” and “Strongly agree (5).” Items in the negative attitude sub-dimensions are scored inversely. The item scores are summed in each subdimension and divided by the number of the items in the subdimension, a value between 1 and 5 is obtained. High scores obtained from the positive attitude sub-dimension indicate that the attitude toward the vaccine is positive. Vaccine negative attitude is calculated after the items in the negative attitude sub-dimension are reversed, and the higher scores indicate a lower negative attitude. Inverse items 1 → 5; 2 → 4; 3 → 3; 4 → 2; It is encoded as 5 → 1 (10).

### Avoidance attitudes from COVID-19

The Avoidance Attitudes from COVID-19 (AA-COVID-19) scale consists of 10 items and is a five-point Likert scale. It has two sub-dimensions, cognitive avoidance, and behavioral avoidance. Behavioral avoidance from COVID-19 includes items such as “avoiding participating in social activities to prevent the disease,” “avoiding taking public transport to prevent getting sick,” “not kissing when greeting people you know,” “not shaking hands when greeting people,” “avoiding using public toilets.” Examples of items from the cognitive avoidance sub-dimension include “Distracting your attention when exposed to news about the disease” and “not reading news about the pandemic.” Expressions in the scale are evaluated as I definitely do not (1), I do not (2), I am undecided (3), I do (3), and I definitely do (5). There is no reverse item on the scale. The item scores are summed in each subdimension and divided by the number of the items in the subdimension, a value between 1 and 5 is obtained. High scores from the sub-dimensions indicate high levels of avoidance in the relevant domain (10).

### Adult ADHD self-report scale symptoms checklist

Adult ADHD Self-Report Scale (ASRS-v1.1) Symptoms Checklist is an 18-item 5-point Likert-type scale that questions ADHD symptoms in adults according to DSM-IV criteria. It is developed by The World Health Organization (WHO). Inattention and HI are two subscales of the ASRS. Each item is scored between 0 and 4. (0–4 = never, rarely, sometimes, often, to very often) Total scores ranged from 0 to 72 (14). In the reliability analysis, the internal consistency of the scale was found to be high (Cronbach's alpha = 0.88). The Cronbach alpha value calculated for the subscales was also found to be high, 0.82 for “attention deficit” and 0.78 for hyperactivity/impulsivity. In addition, the 2-week test-retest consistency, evaluated in 50 subjects, was high (*r* = 0.85 for total scores; *r* = 0.73–0.89 for subscales). The validity and reliability of the Turkish version of the ASRS were developed by Dogan et al. (15).

### Adult ADHD self-report screening scale for DSM-5

Among the valid ADHD screening scales that are currently in use, most of them are calibrated to DSM-IV criteria including the ASRS-v1.1. However, DSM-5 reduced the required number of symptoms from six to five, and the age of onset was updated to seven instead of 12. ASRS-v1.1 was updated according to DSM-5. Adult ADHD Self-Report Screening Scale for DSM-5 (ASRS-5) is a 5-point Likert-type ADHD screening scale consisting of 6 items developed by WHO in line with the DSM-5 diagnostic criteria. Each item is scored between 0 and 4. Total scores ranged from 0 to 24 (16). A validity study was performed (17).

### Patient-health questionnaire-9

The nine-item, one-page patient health questionnaire-9 (PHQ-9) is a screening test for depression with high sensitivity and specificity (88% sensitive, 88% specific if the score is ≥10). Each item is scored between 0 (not at all) and 3 (nearly every day). The total score ranges from 0 to 27. A possible depressive disorder is indicated with values of 10 and above (18). Turkish reliability of the patient health questionnaire-9 was conducted by Sari and colleagues (19). The diagnostic validity of the 9-item PHQ-9 was established in studies involving 8 primary care and 7 obstetrical clinics. PHQ-9 scores >10 had a sensitivity of 88% and a specificity of 88% for major depressive disorder. The reliability and validity of the tool have indicated it has sound psychometric properties. The internal consistency of the PHQ-9 is high.

### State and trait anxiety inventory

It is a 4-point Likert-type self-report scale that includes two separate scales (STAI-I and STAI-II) each of which consists of 20 items and a total of 40 items. High scores indicate a high level of anxiety. State anxiety refers to how the individual feels at a certain moment and under certain conditions, and trait anxiety refers to how he feels regardless of the situation and conditions. The total score on the scale ranges from 20 to 80. Higher scores show higher levels of anxiety and lower scores show lower levels of anxiety (20). Internal consistency coefficients for the scale have ranged from 0.86 to 0.95; test-retest reliability coefficients have ranged from 0.65 to 0.75 over a 2-month interval (21). Test-retest coefficients for this measure in the present study ranged from 0.69 to 0.89. Considerable evidence attests to the construct and concurrent validity of the scale (21). Validity and reliability of the Turkish version of the STAI were carried out by Oner and Le Comte (22).

### Statistical analysis

The statistical power analysis showed that a minimum sample of *n* = 55 would have the assumption of linear multiple regression to achieve 80% power (ß = 0.2) with a 5% significance level (α = 0.05) in a two-tailed test (23). SPSS Version 26.0 was used to analyze the data (IBM Inc. Armonk, NY, USA). The normality test, Kolmogorov Smirnov, was used to examine the data distribution. Descriptive statistics were presented as number and proportion for categorical variables, and non-normal distributed variables as “median (min-max).” Nonparametric statistical methods were used because the data were not normally distributed. The Mann–Whitney *U*-test and The Kruskal–Wallis test were used to compare non-normalized continuous variables. The Chi-Square test, Fisher Exact test, and Fisher-Freeman-Halton Exact Test were used to compare categorical variables. Univariate logistic regression was carried out to examine the impact of variables on the outcome of COVID-19 infection status. Spearman correlations coefficients were used to analyze associations between the independent variables and the outcome. Multiple linear regression with a backward method was applied to analyze factors significantly impacting avoidance of COVID-19. A statistically significant result is defined as a *p*-value of <0.05 (two-tailed).

## Results

### Sociodemographic and clinical characteristics of patients with ADHD

Table 1 shows that there were no significant sex differences in any of the sociodemographic, clinical, or COVID-19 infection or vaccination characteristics among patients.

**Table 1:** Sociodemographic and clinical characteristics of ADHD patients (*n* = 90).

	**Males**	**Female**	**χ^2^**	***p***
	**(*n* = 46)**	**(*n* = 44)**		
Age (median, min-max)	23 (18–42)	23 (18–54)		0.740
Educational status	***n*** **(%)**	***n*** **(%)**	1.374	0.503
High school and lower	15 (32.60)	10 (22.72)		
Bachelor	27 (58.69)	28 (63.64)		
Graduate and higher	4 (8.71)	6 (13.64)		
Vocational status			0.007	0.934
Employed/student	40 (86.95)	38 (86.36)		
Unemployed	6 (13.04)	6 (13.64)		
Marital status			1.152	0.283
Married	9 (19.64)	5 (11.44)		
Single	37 (80.36)	39 (88.56)		
Income level			3.490	0.175
Low	5 (10.88)	10 (22.71)		
Moderate	28 (60.92)	19 (43.19)		
High	13 (28.20)	15 (34.10)		
Medication type			1.356	0.527^a^
Methylphenidate	33 (71.74)	28 (63.66)		
Atomoxetine	1 (2.14)	3 (6.82)		
None	12 (26.12)	13 (29.52)		
COVID-19 infection status				
COVID-19 positive	17 (37.00)	19 (43.25)	0.363	0.547
COVID-19 negative	29 (63)	25 (56.75)		
COVID-19 vaccination			1.084	0.655^a^
Fully vaccinated	36 (78.26)	38 (86.36)		
Incompletely vaccinated	6 (13.04)	4 (9.09)		
Not vaccinated	4 (8.70)	2 (4.55)		

a Fisher Freeman Halton Exact test.

χ^2^, Pearson chi-square value.

### Comparison of patients and controls in terms of clinical and COVID-19 characteristics

Patients and controls did not significantly differ in gender, education, and income level. The median age of patients (23, min-max: 18–54) was significantly lower than the controls (25, min-max: 19–40; *p* = 0.022). ADHD patients' score was higher than controls in inattention, hyperactivity-impulsivity, depression, and trait anxiety measured by ASRS v1.1., and ASRS-5, PHQ-9, and STAI-II, respectively (Table 2).

**Table 2:** Characteristics of patients and controls in terms of clinical and COVID-19 characteristics.

**Characteristics**	**Patients**	**Controls**	**χ^2^**	***p***
	**(*n* = 90)**	**(*n* = 40)**		
Age (median, min–max)	23 (18–54)	25 (19–40)		0.019
Sex	***n*** **(%)**	***n*** **(%)**	0.014	0.907
Male	46 (51.1)	20 (50)		
Female	44(48.9)	20 (50)		
Educational status			2.744	0.098
High school and lower	25 (27.8)	17 (42.5)		
Bachelor and higher	65 (72.2)	23 (57.5)		
Vocational status			0.064	0.8
Unemployed	12 (13.3)	6 (15)		
Employed/student	78 (77.5)	34 (34.5)		
Income level			0.370	0.831
Low	15 (16.7)	5 (12.5)		
Moderate	47 (52.2)	22 (55.00)		
High	28 (31.1)	13 (32.5)		
Inattention	24 (4–36)	9.50 (3–17)	—	<0.001
Hyperactivity impulsivity	19 (6–36)	9 (5–15)	—	<0.001
ASRS-5	15 (4–24)	8 (2–11)	—	<0.001
PHQ-9	15 (2–38)	7 (0–24)	—	<0.001
STAI-I	40 (32–59)	38.5 (32–46.7)	—	0.055
STAI-II	43 (27–61)	38 (12–48)	—	<0.001
P-COVID-19			—	
Dangerousness	4.33 (2.67–5)	4.33 (2.67–5)	—	0.436
Contagiousness	3.91 (2–5)	3.50 (1.75–5.00)	—	0.038
ATV-COVID-19			—	
Positive attitudes	3.5 (1–5)	3 (1–5)	—	0.024
Negative attitudes	3.60 (1.4–4.80)	3.10 (1.8–5)	—	0.192
AA-COVID-19			—	
Cognitive avoidance	2.50 (1–4.40)	2.00 (1–4)	—	0.008
Behavioral avoidance	3.90 (1.4–5)	3.2 (2.20–5.00)	—	0.053
COVID-19 infection status	*n* (%)	*n* (%)	0.638	0.424
COVID-19 positive	36 (40)	19 (47.5)		
COVID-19 negative	54 (60)	21(52.5)		
COVID-19 vaccination			4.815^b^	0.071^a^
Fully vaccinated	74 (82.22)	37 (92.5)		
Incompletely vaccinated	10 (11.11)	0 (0)		
Not vaccinated	6 (6.67)	3 (7.50)		

a Fisher-Freeman-Halton Exact test, other comparisons between categorical variables were carried out with Chi-square.

b Fisher's exact test value: 5.275. Continuous data were compared with Mann–Whitney U.

STAI-I & STAI-II, state and trait anxiety inventory I-II; PHQ-9, patient health questionnaire-9; ATV-COVID19, attitudes toward the COVID-19 vaccine; P-COVID-19, perception of the COVID-19 disease; AA-COVID-19, the avoidance attitudes from COVID-19.

Regarding COVID-19 measures, ADHD patients were more likely to have a perception of COVID-19 as contagious, a more positive attitude toward the COVID-19 vaccine, and greater cognitive avoidance (i.e., avoiding thinking about COVID-19) than controls. There were no significant group differences in perception of COVID-19 as dangerous and behavioral avoidance of COVID-19. The majority of the patients and controls had been fully vaccinated. There was no significant group difference in infection status (Table 2).

### Characteristics of patients by COVID-19 positive or negative testing

When patients were split into two groups, the first group was composed of patients who have ever tested positive for COVID-19, and the second group was composed of those who have never tested positive for COVID-19. There was no significant difference in terms of any sociodemographic and clinical variables between these two groups of patients (Table 3).

**Table 3:** Characteristics of patients by COVID-19 positive or negative testing.

**Characteristics**	**Test positive**	**Test negative**	***p***
**(median, min-max)**	**for COVID-19**	**for COVID-19**	
	**(*n* = 36)**	**(*n* = 54)**	
Age	21.5 (18–47)	23 (18–54)	0.145
ASRS total score	45.5 (19–64)	42 (7–72)	0.783
Inattention	26 (8–34)	24 (4–36)	0.465
Hyperactivity-impulsivity	18.5 (9–30)	19.50 (6–36)	0.717
ASRS-5	15 (5–22)	15 (4–24)	0.588
PHQ-9	14 (3–38)	17 (2–27)	0.701
STAI-I	41 (34–48)	40 (32–59)	0.088
STAI-II	43 (27.5–54)	44(27–61)	0.941
P—COVID-19
Dangerousness	4.67 (3–5)	4 (2.67–5)	0.051
Contagiousness	3.9 (2–5)	3.88 (2–4.75)	0.947
ATV-COVID-19
Positive attitudes	3.5 (1–5)	3.5 (1–5)	0.601
Negative attitudes	3.4 (1.4–4.8)	3.6 (2–4.6)	0.970
AA-COVID-19			
Cognitive avoidance	2.6 (1–4.2)	2.4 (1–4.4)	0.533
Behavioral avoidance	4 (1.6–5)	3.8 (1.4–4.8)	0.378
Sex	***n*** **(%)**	***n*** **(%)**	0.722
Male	17 (47.22)	29 (53.70)	
Female	19 (52.78)	25 (46.30)	
Educational status			0.311
High school and lower	12 (33.33)	13 (24.07)	
Bachelor	22 (61.11)	33 (61.11)	
Graduate and higher	2 (5.56)	8 (14.82)	
Vocational status			1.000
Employed/student	31 (86.11)	47 (87.03)	
Unemployed	5 (13.89)	7 (12.97)	
Marital status			0.722
Married	5 (13.89)	9 (16.66)	
Single	31 (86.11)	45 (83.34)	
Income level			0.462
Low	4 (11.11)	11 (20.37)	
Moderate	21 (58.33)	26 (48.14)	
High	11 (30.56)	17 (31.49)	
Medication type			0.319^a^
Methylphenidate	24 (66.67)	37 (68.51)	
Atomoxetine	0 (0.00)	4 (7.40)	
None	12 (33.33)	13 (24.09)	
COVID-19 vaccinated			0.909^a^
Fully vaccinated	30 (83.33)	44 (81.48)	
Incompletely vaccinated	5 (13.88)	5 (9.26)	
Not vaccinated	1 (2.79)	5 (9.26)	
Presence of ADHD medication			0.921
No	14 (25.9)	9 (25.0)	
Yes	40 (74.1)	27 (75)	

a Fisher-Freeman-Halton Exact test, other comparisons between categorical variables were carried out with Chi-square, Continuous data were compared with Mann–Whitney U.

STAI-I & STAI-II, state and trait anxiety inventory I-II; PHQ-9, patient health questionnaire-9; ATV-COVID19, attitudes toward the COVID-19 vaccine; P-COVID-19, perception of the COVID-19 disease; AA-COVID-19, the avoidance attitudes from COVID-19.

### Factors affecting the status of COVID-19 infection

Univariate logistic regression analyses were performed in patients and controls to determine which factors impacted positive or negative COVID-19 infection status. The analyses showed that there was no significant impact of any of the sociodemographic and clinical variables on infection status.

To investigate the effect of the type of ADHD medication on COVID-19 status in patients, a Chi-square analysis (COVID-19 infection status X the presence of ADHD medication) was carried out. No significant group differences were found between patients who were on an ADHD medication and those who were not [*X*^2^ (1, *N* = 90) = 0.923, *p* = 0.337]. Mann–Whitney *U*-test revealed that behavioral avoidance did not significantly differ between ADHD patients on medication and patients, not on medication (*p* = 0.339).

### Correlations of clinical characteristics, COVID-19-related factors, and behavioral avoidance of COVID-19

Spearman correlation coefficients were used to test the associated factors with behavioral avoidance of COVID-19. Findings were summarized in Table 4. Perception of COVID as dangerous and contagious, attitudes toward COVID-19 vaccine, depression score, and age were significantly correlated with behavioral avoidance (Table 4).

**Table 4:** Correlations of clinical characteristics, COVID-19-related factors, and behavioral avoidance of COVID-19.

	**P-COVID-19 as dangerous**	**P-COVID-19 as contagious**	**Cognitive AA-COVID-19**	**Behavioral AA-COVID-19**	**Positive ATV-COVID19**	**Negative ATV-COVID19**	**Hyperactivity/ Impulsivity**	**Inattention**	**STAI-I**	**STAI-II**	**PHQ-9**	**Age**
P-COVID-19 as dangerous	1											
P-COVID-19 as contagious	0.382^**^	1										
Cognitive AA-COVID-19	−0.043	−0.031	1									
Behavioral AA-COVID-19	0.349^**^	0.297^**^	0.052	1								
Positive ATV-COVID19	0.192*	0.242^**^	−0.094	0.284^**^	1							
Negative ATV-COVID19	0.227^**^	0.295^**^	−0.045	0.235^**^	0.685^**^	1						
Hyperactivity/ impulsivity	0.067	0.302^**^	0.354^**^	0.077	0.245^**^	0.085	1					
Inattention	0.093	0.223*	0.288^**^	0.03	0.226^**^	0.179*	0.769^**^	1				
STAI-I	0.003	0.129	−0.064	0.011	0.139	0.074	0.241^**^	0.132	1			
STAI-II	0.158	0.173*	0.227^**^	0.023	0.086	−0.093	0.459^**^	0.476^**^	0.245^**^	1		
PHQ-9	0.185*	0.200*	0.240^**^	0.151	0.15	0.064	0.589^**^	0.653^**^	0.016	0.497^**^	1	
Age	0.061	0.191*	−0.085	0.203*	0.217*	0.214*	−0.092	−0.186*	−0.077	−0.052	−0.196*	1

** Correlation is significant at the 0.01 level and * at the 0.05 level (2-tailed).

STAI-I & STAI-II, state and trait anxiety inventory I-II; PHQ-9, patient health questionnaire-9; ATV-COVID19, attitudes toward the COVID-19 vaccine; P-COVID-19, perception of the COVID-19 disease; AA-COVID-19, the avoidance attitudes from COVID-19.

### Factors affecting behavioral avoidance of COVID-19

Factors that were associated with behavioral avoidance using a cut-off of the *p*-value of 0.10 in Spearman correlation analysis were perception of COVID-19 as dangerous and contagious, positive and negative attitudes toward COVID-19 vaccine, depression score, and age. These were entered into multiple linear regression which was performed to predict behavioral avoidance of COVID-19 (*n* = 130). The final linear regression model included the perception of COVID-19 as dangerous and a positive attitude toward the COVID-19 vaccine as variables that significantly impact the outcome of behavioral avoidance. The model explained 17% of the variance in the whole group [*R*^2^ = 0.17, *F* (2) = 13.189, *p* < 0.0001; Table 5).

**Table 5:** Linear regression analysis exploring factors that impact behavioral avoidance of COVID-19.

**Factors**	**Coefficient (B)**	**SE**	**95% CI**	***p***
Perception of COVID-19 as dangerous	0.346	0.092	0.164–0.528	0.000
Positive attitudes toward the COVID-19 vaccine	0.166	0.059	0.048–0.164	0.006

Model F(2) = 13.189, p < 0.0001, R^2^ = 0.17.

## Discussion

This study looked at COVID-19 infection, COVID-19 vaccine, and the factors that affect COVID-19 infection and behavioral avoidance of COVID-19. Our research was driven by the few and inconsistent findings in the literature regarding COVID-19 risk in ADHD, lack of data on vaccine status in ADHD, and scarcity of data to guide recommendations specified for this group. We think that the gap must be filled to know where to intervene to support ADHD patients by adhering to protective measures. Contrary to our expectations, the COVID-19 infection status, COVID-19 vaccine status, perception of COVID-19 as dangerous, and behavioral avoidance of COVID-19 in adults with ADHD were not significantly different compared with controls. No demographic and clinical factors significantly affected the COVID-19 infection status. Avoidance of COVID-19 was significantly correlated with age, perception of COVID-19 as dangerous and contagious, and a positive perception of the COVID-19 vaccine. After adjustment of other factors, a positive perception of the COVID-19 vaccine and perception of COVID-19 as dangerous were the two factors significantly affecting behavioral avoidance of COVID-19 in ADHD. Approximately four-fifths of the patients were fully vaccinated as recommended by national and global health organizations.

### COVID-19 infection status in ADHD

COVID-19 infection status did not significantly differ among patients and controls which was not in line with some studies (2, 3) but corroborated with others (7). The discrepancy may firstly be explained by differences in the timing of the studies. Studies by Merzon and Wang analyzed the health records during the first wave of the pandemic when several uncertainties exist about COVID-19 (2, 3). We collected data much later and in the second half of the pandemic. The disease got to be recognized as a contagious and dangerous public health condition with known consequences. Mitigation efforts including the positive attitudes toward the vaccine and the high vaccination rate may have provided adequate protection that individual risk-taking behaviors were subdued and any group differences in COVID-19 status may have been repressed. This raises a very important implication which is addressing the dangerousness and contagiousness of the COVID-19 infection and adequately informing individuals with ADHD and possibly other mental disorders may reciprocate despite the increased risk of infection in the first place. Secondly, differences in methodology may account for different findings. The first study assessed the ADHD rate among people from the general population while we looked into the COVID-19 rate among ADHD people. Thirdly, ADHD can be considered a mental disorder with neuropsychological disturbances that may manifest in three levels: behavior, cognitive functions, and separate component of cognitive functions (24). Despite hyperactivity-impulsivity, attentional control difficulties, and a complex system of executive function disturbances including neurodynamic deficits (25), ADHD itself may provoke no kind of mental disturbance in sense of perception of the danger of this disease and protection of it by avoidance behaviors and receiving the vaccine.

Different cultural contexts with variances in pandemic management may affect the findings (2). Fourthly, the relatively higher educational status of the patients in our study may have additionally contributed to the findings. Education was reported to be a factor that predicted risk-mitigation behaviors against COVID-19 (26). The characteristic of a college degree and above education was shown to be a protective factor for COVID-19 infection (27). Besides inattention (3) having fewer worries and a diminished level of concern were proposed to be other reasons for not taking the necessary precautions (7).

Our findings are consistent with a study in children where researchers showed that children with ADHD were not more likely to experience COVID-19 infection. This study followed a similar methodology to ours and demonstrated the COVID-19 rate among ADHD and controls (7). Another study exhibited no correlations between ADHD and population size infection and mortality rates from coronavirus. Interestingly, this study showed that recovery rates (recovery-population ratio) rise with the prevalence of ADHD. ADHD might provide an evolutionary advantage in coping with the disease like the non-dominant gene that helps to compete with malaria in sickle-cell disease (9).

We found that no demographic or clinical factors significantly impacted COVID-19 infection status. COVID-19 positive test status did not differ between treated and untreated ADHD patients whereas this finding did not corroborate with two studies that demonstrated that treated ADHD subjects had a lower risk for COVID-19 than untreated ADHD (2, 28). The discrepancy could be explained by the different study populations. The first study included both children and adults and the latter study included only children (28). Another explanation could be different presentations of ADHD during childhood and adulthood. HI symptoms are more dominant during childhood whereas more subtle or subsided during adulthood. It is expected that untreated children may participate in activities that may raise the risk of COVID-19 (e.g., running around, leaving their seats) (11, 29). On the other hand, young adults are expected to have more behavioral limits and a stronger awareness of the idea of social distance therefore could be more complying with precautions than children (30).

We found males and females with ADHD had similar rates of COVID-19 infection and vaccination rates whereas a positive association of COVID-19 infection with the male gender was demonstrated in the study by Merzon et al. (2). On the contrary, Wang and colleagues demonstrated among people who tested positive for COVID-19, women with ADHD had higher odds of COVID-19 infection than males with ADHD and who were diagnosed with ADHD within the year before the study was carried out. The data regarding the gender, ADHD, and COVID-19 infection risk appear inconsistent.

### Behavioral avoidance in ADHD patients

Avoidance behaviors are one of the COVID-19 risk mitigation measures (11) which are very important in the management of and fight against the pandemic. They include physical distancing, staying at home, avoiding close contact like shaking hands or kissing, and avoiding participating in social activities and public places. We found that significant factors in the behavioral avoidance outcome were the perception of COVID-19 as dangerous and positive attitudes toward the vaccine but not any clinical characteristics of ADHD symptoms, state and trait anxiety and depression.

Our findings on behavioral avoidance corroborate a very recent study that investigated the risk-mitigation practices in youth with ADHD. Of five different groups of disorders including ADHD, only anxiety disorders were associated with avoidance behaviors (which included avoiding groups, indoor settings, and other people's homes) (11). Cognitive avoidance which is known to be an unhealthy coping mechanism was higher in ADHD patients compared with controls.

### Vaccination tendencies and actual practice in ADHD adults

Our findings increased the knowledge base by demonstrating that the acceptability of the COVID-19 vaccine and actual practice of receiving the vaccine was endorsed in ADHD patients with no significant difference from healthy controls. To the best of our knowledge, this finding has not been previously reported in the literature. Approximately four-fifths of the patients had the full vaccination as recommended by the national and global health bodies. Although there was a higher number of patients who had incomplete COVID-19 vaccination compared to controls, this did not reach statistical significance. In a study with children with ADHD, one-fourth of caregivers of ADHD children were hesitant to vaccinate their children (31).

### Strengths and limitations

This study expands our knowledge on the perceptions of COVID-19 and its vaccine and the avoidance behaviors which are important to mitigate the risk of infection. To the best of our knowledge, the data have not been reported before in the literature. Nevertheless, our study has limitations. First, it should be taken into account that data were gathered throughout various pandemic phases without distinguishing the different phases of the pandemic although there was a match and balance between patients and controls. Second, the findings' generalizability could be hampered by the relatively small sample size. Third, the sample of our study may not reflect the perceptions and behaviors of ADHD patients from all cultural backgrounds. Additionally, healthy controls were selected based on their self-report of having no psychiatric disorders and medications, which is a further limitation. The age range in our study was 18–54 so the findings may not represent older adults with ADHD. Most of the ADHD patients were contacted by phone to invite to the study and only those who were willing to participate were recruited; although this may pose a selection bias, there were no significant differences in age, gender, and education in those who participated and those who did not. Only one patient had a chronic medical disorder and only two patients had substance use disorder in our sample. Therefore, examining the role of comorbid medical diseases except for depression and anxiety on COVID-19 infection was not possible.

Those with ADHD may exhibit different stress symptom profiles compared with typically developing subjects (29). These differences may necessitate measuring anxiety and depression symptoms to develop targeted strategies (30). COVID-19-related perceptions may also play a role in explaining the link (32).

To conclude, this study looked at COVID-19 infection and vaccine status and the factors that affect COVID-19 infection status and behavioral avoidance of COVID-19 among adults with ADHD. Our research was driven by the few and inconsistent findings, lack of data on vaccine status, and scarcity of data to guide recommendations specified for this group. We think the gap must be filled to intervene and support ADHD patients to adhere to protective measures. To the best of our knowledge, COVID-19 vaccine acceptability among ADHD has not been reported before in the literature. Approximately four-fifths of the patients were fully vaccinated as recommended by national and global health organizations. Contrary to our expectations, the COVID-19 infection and vaccine status, perception of COVID-19 as dangerous, and behavioral avoidance of COVID-19 did not differ from controls. No factor significantly affected the COVID-19 infection status. After adjustment of possible factors, a positive perception of the COVID-19 vaccine and perception of COVID-19 as dangerous were the two factors significantly affecting behavioral avoidance of COVID-19 but not the clinical characteristics of ADHD symptoms, state-trait anxiety, and depression. Our findings have increased the knowledge base showing that the COVID-19 vaccine is acceptable and the actual practice of receiving the vaccine is endorsed among ADHD patients.

Our message for practice would be to take into account not only the core symptoms and the comorbidities of the disorder but also the perception of the disease among patients. ADHD itself may provoke no kind of mental disturbance in sense of perception of the danger of this disease.

Further studies to better understand the mechanisms of mental illness and COVID-19 infection are in need (33). A comparison between the early findings and later findings on COVID-19 risk among ADHD individuals during the course of the pandemic is warranted.

## Data availability statement

The datasets presented in this article are not readily available because of confidentiality restrictions on the patient data. Requests to access the datasets should be directed to OK, drozgekilic@gmail.com.

## Ethics statement

The studies involving human participants were reviewed and approved by Bezmialem Vakif University Clinical Research Ethics Committee. The patients/participants provided their written informed consent to participate in this study.

## Author contributions

OK, MB, ED, and IK contributed to the conception and design of the study. MS-B and SK-E organized the database. OK, MB, MS-B, and SK-E carried out data collection. OK performed the statistical analysis and wrote the first draft of the manuscript. OK, MB, GG, SY, and ED wrote sections of the manuscript. All authors contributed to manuscript revision, read, and approved the submitted version.

## Conflict of interest

The authors declare that the research was conducted in the absence of any commercial or financial relationships that could be construed as a potential conflict of interest.

## Publisher's note

All claims expressed in this article are solely those of the authors and do not necessarily represent those of their affiliated organizations, or those of the publisher, the editors and the reviewers. Any product that may be evaluated in this article, or claim that may be made by its manufacturer, is not guaranteed or endorsed by the publisher.
